# *In Vitro* Assessment of the Cercaricidal Activity of *Sida acuta* Burm. F. and *Sida rhombifolia* Linn. (Malvaceae) Hydroethanolic Extracts, Cytotoxicity, and Phytochemical Studies

**DOI:** 10.1155/2022/7281144

**Published:** 2022-01-10

**Authors:** Ulrich Membe Femoe, Joseph Bertin Kadji Fassi, Hermine Boukeng Jatsa, Yannick Leonel Tchoffo, David Carel Amvame Nna, Blaise Cédric Kamdoum, Steven Collins Njonte Wouamba, Billy Toussie Tchegnitegni, Bonaventure Tchaleu Ngadjui, Norbert Sewald, Bruno Ndjakou Lenta, Louis-Albert Tchuem Tchuente, Theophile Dimo

**Affiliations:** ^1^Department of Animal Biology and Physiology, Faculty of Science, University of Yaounde I, P.O. Box 812, Yaounde, Cameroon; ^2^Centre for Schistosomiasis and Parasitology, P.O. Box 7244, Yaounde, Cameroon; ^3^Department of Organic Chemistry, Faculty of Science, University of Yaounde I, P.O. Box 812, Yaounde, Cameroon; ^4^Department of Chemistry, Higher Teacher Training College, University of Yaounde I, P.O. Box 47, Yaounde, Cameroon; ^5^Department of Organic and Bioorganic Chemistry, Faculty of Chemistry, Bielefeld University, P.O. BOX 100131, D-33501, Bielefeld, Germany

## Abstract

Despite the global efforts, schistosomiasis remains a public health problem in several tropical and subtropical countries. One of the major challenges in the fight against schistosomiasis is the interruption of the parasite life cycle. Here, we evaluated the anticercarial, cytotoxicity, and phytochemical profiles of *Sida acuta* (HE*Sa*) and *Sida rhombifolia* (HE*Sr*) hydroethanolic extracts (Malvaceae)*. Schistosoma mansoni* cercaria was collected from fifteen *Biomphalaria pfeifferi*-infected snails. Twenty-five cercariae were incubated in duplicate with different concentrations (31.25–1,000 *μ*g/mL) of HE*Sa* or HE*Sr*. The cercaria viability was monitored at 30 min time intervals for 150 min, and the concentration-response curve of each plant extract was used to determine their respective lethal concentration 50 (LC_50_). Additionally, the cytotoxicity profile of each plant extract was evaluated on the Hepa 1–6 cell line at a concentration range of 15.625–1,000 *µ*g/mL using the WST-8 assay method and its inhibitory concentration 50 (IC_50_) was calculated. Moreover, phytochemical characterization of each plant extract was carried out by HPLC-MS. Both extracts exhibited cercaricidal activity in a time- and concentration-dependent manner. At 30 min time point, HE*Sa* (LC_50_ = 28.41 ± 3.5 *µ*g/mL) was more effective than HE*Sr* (LC_50_ = 172.42 ± 26.16 *µ*g/mL) in killing *S. mansoni* cercariae. Regarding the cytotoxicity effect of both extracts, the IC_50_ of HE*Sa* (IC_50_ = 109.67 *µ*g/mL) was lower than that of HE*Sr* (IC_50_ = 888.79 *µ*g/mL). The selectivity index was 3.86 and 5.15 for HE*Sa* and HE*Sr*, respectively. Fifteen compounds were identified from HE*Sa* and HE*Sr* after HPLC-MS analysis. N-Feruloyltyramine, a polyphenol, and thamnosmonin, a coumarin, were identified in both extracts. HE*Sa* and HE*Sr* displayed cercaricidal activity and were not toxic on Hepa 1–6 cell line. Based on the selectivity index of these extracts, *S. rhombifolia* extract could be more effective on S. mansoni cercariae than S. acuta extract. This study could provide baseline information for further investigations aiming to develop plant-based alternative drugs against *S. mansoni*.

## 1. Introduction

Schistosomiasis, also known as bilharzia, is an infectious disease caused by trematodes flatworms of the genus *Schistosoma.* It is known as one of the most prevalent tropical diseases worldwide. It is estimated that 229 million people required preventive treatment in 2018 and close to 800 million are at risk of infection [1]. After an infected person releases *Schistosoma* eggs into the water by defecation or urination, the ripe miracidia hatch out and invade the intermediate host freshwater snail where they form sporocysts. They then matured to give cercariae that emerges from snails and swims to penetrate the skin of humans and/or animals that are definitive hosts [2]. Therefore, to control schistosomiasis, the life cycle of the parasite can be interrupted by killing cercariae and miracidia [3–5]. The use of chemical compounds to control the aquatic snails, cercariae, or miracidia is not recommended because of their adverse effects on the environment [6]. A large number of plant families with potential schistosomicidal activity have been identified through plant screening, which represents a continuous effort to find new bioactive molecules. The anti-cercarial activities of several plants such as *Rauwolfia vomitoria* [7], *Nigella sativa* [8] *Balanites aegyptica*, *Azadirachta indica*, *Nauclea latifolia*, *Morinda lucida*, *Phyllanthus amarus*, *Vernonia amygdalina* [9], and *Ozoroa pulcherrima* [10] have been reported. In several countries, *Sida L*. (Malvaceae) has been used for centuries in traditional medicines for the prevention and treatment of different diseases such as diarrhea, dysentery, gastrointestinal and urinary infections, cancer, malaria, and helminth infections [11–18]. Moreover, during the last decade, Jatsa et al. [19–21] have demonstrated the *in vitro* and *in vivo* anti-schistosomal potency of *Sida pilosa*. In addition, previous investigations on two of the plants of the genus *Sida*, namely *Sida acuta* and *Sida rhombifolia*, reported their anthelmintic and larvicidal activities [22–24]. Considering the above, this study was therefore carried out to evaluate the i*n vitro* cercaricidal activity of *S. acuta* and *S. rhombifolia* hydroethanolic extracts against *Schistosoma mansoni* cercaria, their cytotoxicity profile, and determine their phytoconstituents.

## 2. Materials and Methods

### 2.1. Collection, Preparation, and Extraction Process of Plant Specimens

**Figure 1:**
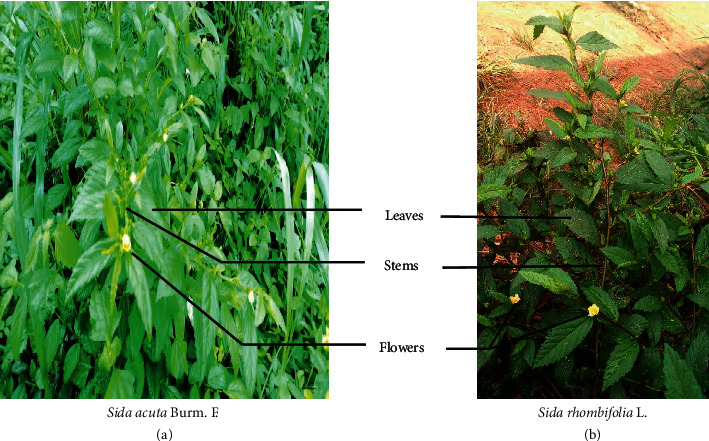
Aerial parts of (a) *Sida acuta* and (b) *Sida rhombifolia*.

The whole plant of *S. acuta* (Figure 1(a)) was collected in February 2017 at Nkoemvone near Ebolowa in the South Region while the aerial parts of *S. rhombifolia* (Figure 1(b)) were harvested in March 2017 at Bazou, a village of Ndé division in the West region of Cameroon. The plant specimens were identified and authenticated at Cameroon's National Herbarium under a voucher specimen HNC n°46188 (*S. acuta*) and HNC n°20113 (*S. rhombifolia)*. Each plant species was air-dried for 2 weeks, and the dried plant materials were ground into powder. Exactly 400 g of each pulverized plant material was macerated with 800 mL of ethanol/water mixture (70/30) at room temperature for 48 h. The macerates were filtered, and the filtrate was concentrated using a rotary evaporator (Buchi Rotavapor, *R* 200) under reduced pressure at 40°C. The crude extract was finally air-dried, and the yield of obtained hydroethanolic extracts of *S. acuta* (HE*Sa*) and *S. rhombifolia* (HE*Sr*) was calculated.

### 2.2. *In Vitro* Cercaricidal Activity of the Plant Extracts

#### 2.2.1. Snails Infection and Preparation of the Cercarial Suspension

Schistosome cercariae were obtained from experimentally infected juvenile *Biomphalaria pfeifferi* snails that were maintained in the snail laboratory at the Centre Schistosomiasis and Parasitology of Yaoundé, Cameroon, under standard laboratory conditions of 18–26°C. Briefly, each snail was exposed for 12 hours to 4–5 miracidia obtained after hatching of eggs isolated from *S.-mansoni-*infected mice liver. Four weeks later, 14 infected snails, known to be shedding cercariae, were pooled into a glass Beaker with 20 mL of distilled water and allowed to shed cercariae by exposing them to artificial light for 60 min. After the exposition time, snails were removed from the beaker and 100 *μ*L of the cercariae suspension was transferred on three microscope slides. A volume of 100 *µ*L of Lugol was added on each slide to stain and fix cercariae, and the slides were transferred onto a light microscope to estimate the number of cercariae per 100 *µ*L, and the volume of the cercariae suspension containing approximately 25 viable cercariae was determined [7, 9].

#### 2.2.2. In *Vitro* Cercaricidal Assay

The concentrations ranging from 1,000 to 31.25 *µ*g/mL are widely used to evaluate the cercaricidal activity of medicinal plant crude extracts [7, 9]. According to this protocol, serial dilutions (1,000, 500, 250, 125, 62.5, and 31.25 *μ*g/mL) of each of the test plant extract were prepared using distilled water as diluent. A volume of 1 mL of each dilution was added into a well of a 24-well culture plate. Niclosamide-olamide 5% (1 *µ*g/mL; Jiangsu Aijin Agrochemical Co. Ltd., China) was used as a reference control. Approximately 25 cercariae were pipetted into each of the wells. Cercaria mortality was observed for 150 min at 30 min intervals since the infectivity of cercariae is known to be rapidly lost after 12 h [25]. The bottom of each well was observed at 4X magnification using an inverted microscope and cercariae survival and mortality were recorded at specific 30 min time intervals (30, 60, 90, 120, and 150 min). Cercariae were presumed dead when they stopped movement and sank or their tail was detached [26]. All experiments were carried out in duplicate and repeated twice. The viability percentage and the lethal concentration 50 (LC_50_) values of plant extracts on schistosome cercariae were calculated at each time point.

### 2.3. Cytotoxicity Assay of the Extracts against Hepatocyte Cell Lines

Cytotoxicity of *S. acuta* and *S. rhombifolia* extracts was investigated on C57/L mouse melanoma liver cells line (*Hepa 1-6, ATCC CRL-1830*) using WST-8-based assay as previously described by Murata et al. [27]. The cells were cultured in high-glucose Dulbecco's Modified Eagle Medium (DMEM) with pyruvate and L-glutamine (Gibco, Life Technologies, USA), supplemented with 10% (v/v) fetal bovine serum (FBS) heat-inactivated (Serana, Australia) and 1% (v/v) penicillin/streptomycin (Gibco, Life Technologies, USA). The cells were grown in a growth medium at 37°C in a 95% air, 5% CO_2_-humidified incubator. Monolayer cultures reaching a confluence between 80 and 90% were detached using trypsin solution (Sigma Aldrich, Germany) and calibrated using a cell counter chamber (Fast Read 102). The calibrated cell suspension was seeded into 96-well tissue culture microtiter plates at a density of 1 × 10^4^ cells per well and incubated overnight at 37°C in a 5% CO_2_ incubator for cell adhesion. Following incubation, the medium was removed from the cells and replaced with a fresh one followed by the addition of extracts at different concentrations (1,000, 500, 250, 125, 62.5, 31.25, and 15.625 *µ*g/mL). Control wells with cells only were added, and the plates were incubated for 24 h in the same culture conditions. After incubation, cell viability was measured by mitochondrial activity in reducing 2-(2-methoxy-4-nitrophenyl)-3-(4-nitrophenyl)-5-(2,4disulfophenyl)-2H-tetrazolium monosodium salt to formazan using the cell counting kit-8 (WST-8, Abcam, ab228554, UK) according to the manufacturer instructions. After 4 hours of incubation, optical density was measured at 450 nm using a Dynex MRX TC II microplate reader (Dynex Technologies, USA). The results were expressed as a percentage growth of the control cells and the inhibitory concentration 50 (IC_50_) values were calculated as the concentration of the resulting in 50% reduction of absorbance compared to untreated cells. Tests were carried out in duplicate, and each experiment was repeated three times.

### 2.4. Selectivity Index

In the present study, the degree of selectivity of each hydroethanolic plant extract is expressed as the ratio of the IC_50_ obtained for the cell line to the LC_50_ for *S. mansoni* cercariae.

(1) $$\begin{array}{c} \text{SI}=\frac{\text{IC}50\text{ of extract in cell line}}{\text{LC}50\text{ of the same extract in S. mansoni cercariae}}. \end{array}$$

### 2.5. Phytochemical Analysis

#### 2.5.1. Phytochemical Screening

The hydroethanolic extracts of *S. acuta* and *S. rhombifolia* were subjected to qualitative chemical tests to identify phytochemical constituents. The screening of alkaloids, anthraquinones, and cardiac glycosides was performed by Mayer's test, Bontrager's test, and the Keller–Killiani's test, respectively. Ferric chloride (FeCl3) test was used for the identification of phenols and tannins, Fehling's test for reducing sugars, foam test for saponins, and Liebermann–Burchard's test for steroids. The presence of flavonoids, lipids, and terpenoids was performed using the ammonia test, the grease spot test, and Salkowski's test, respectively [28].

#### 2.5.2. Qualitative Determination of Compounds Using LC-DAD-(HR) ESI-MS

*Sample Preparation*. HE*Sa* and HE*Sr* were dissolved in HPLC grade methanol at a concentration of 5 mg/mL and then filtrated through a syringe-filter-membrane. Aliquots of 5 *µ*L were injected into the UPLC-DAD/MS Dionex Ultimate 3000 HPLC (Germany).

*HPLC-MS Conditions*. High-resolution mass spectra were obtained with a quadrupole-time-of-flight (QTOF) spectrometer (Bruker, Germany) equipped with a HESI source. The spectrometer was operated in positive mode (mass range: 100–1,500, with a scan rate of 1.00 Hz) with automatic gain control to provide high-accuracy mass measurements within 0.40 ppm deviation using Na formate as calibrant. The following parameters were used for experiments: spray voltage of 4.5 kV and the capillary temperature of 200°C. Nitrogen was used as sheath gas (10 L/min). The spectrometer was attached to an Ultimate 3000 UHPLC system (Thermo Fisher, USA) consisting of an LC-pump, Diode Array Detector (DAD; *λ*: 190–600 nm), autosampler (injection volume: 10 *μ*L), and a column oven (40°C). The separations were performed using a Synergi MAX-RP 100A (50 × 2 mm, 2.5 *μ* particle size) with a H2O (+0.1% HCOOH) (A)/acetonitrile (+0.1% HCOOH) (B) gradient (flow rate: 500 *μ*L/min and injection volume: 5 *μ*L). Samples were analyzed using a gradient program as follows: A linear gradient starting with 95% eluent A isocratic over 1.5 min to linear gradient with 100% eluent B over 6 min; 2 min after, the system returned to its initial condition (90% of eluent A) within 1 min and was equilibrated for 1 min.

*Identification of Peaks*. Identification of all constituents was performed by UPLC-DAD-MS analysis and by comparing the UV and MS spectra and MS/MS fragmentation of the peaks in the samples with those of data reported in the literature of the SciFinder, NIST/EPA/NIH Mass Spectral Library (NIST 14), and Mass Bank of North America (MoNA) databases.

### 2.6. Statistical Analysis

Graph drawing and statistical analysis were performed using GraphPad Software version 8.01 (GraphPad Software, San Diego, USA). The results were expressed as means ± SEM, and Student's *t*-test and two-way ANOVA followed by Tukey multiple comparison post-test were used to determine the significance of differences between mean values. A *p*-value less than 0.05 was considered statistically significant.

## 3. Results

### 3.1. Cercaricidal Activity of Plant Extracts

**Figure 2:**
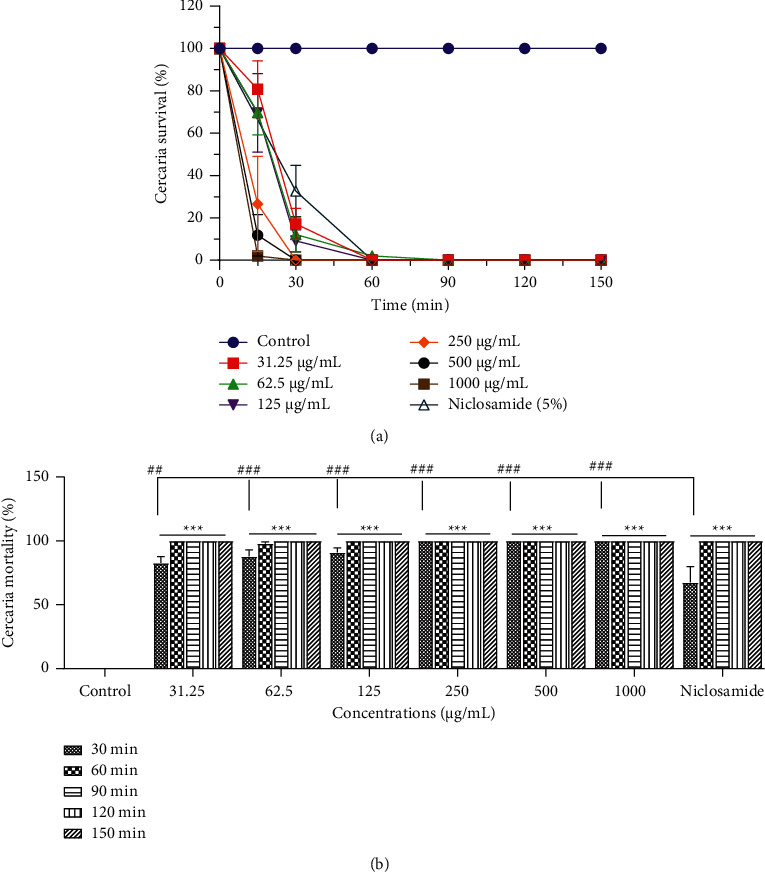
Effect of *Sida acuta* hydroethanolic extract on *Schistosoma mansoni* cercariae viability (a) and mortality rate (b). All bars are expressed as mean ± SEM. ^*∗*^*p* < 0.05 and  ^*∗∗∗*^*p* < 0.001, significantly different from controls (distilled water). ^#^*p* < 0.05 and  ^###^*p* < 0.001, significantly different from reference control (niclosamide).

**Figure 3:**
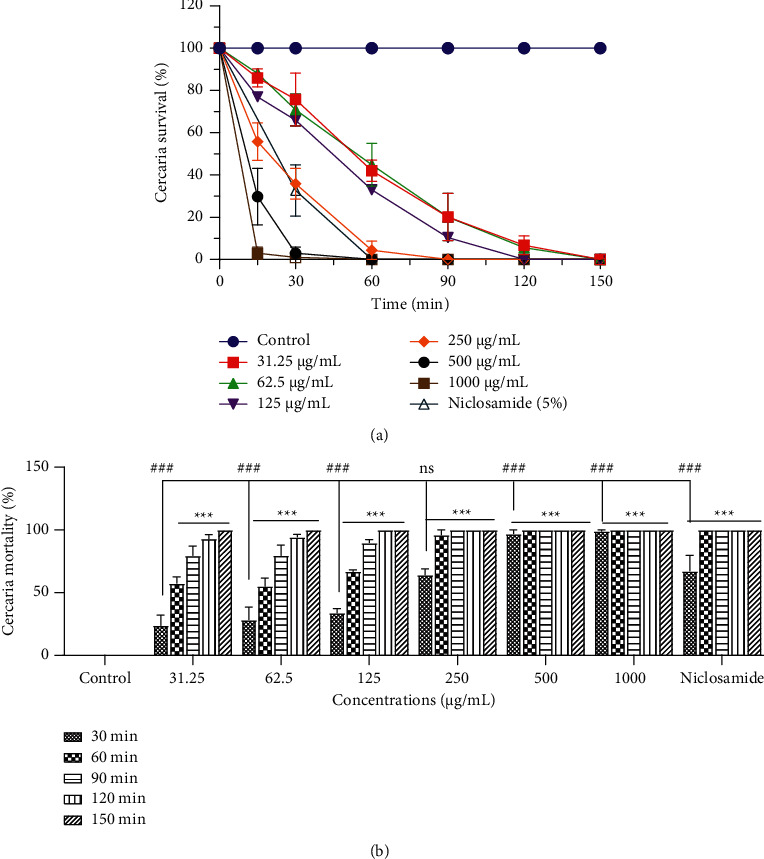
Effect of *Sida rhombifolia* hydroethanolic extract on *Schistosoma mansoni* cercariae viability (a) and mortality rate (b). All bars are expressed as mean ± SEM. ^*∗*^*p* < 0.05 and  ^*∗∗∗*^*p* < 0.001, significantly different from controls (distilled water). ^###^*p* < 0.001, significantly different from reference control (niclosamide).

The mortality of *S. mansoni* cercariae following *in vitro* exposure to different concentrations of *S. acuta* and *S. rhombifolia* extracts was both time- and concentration-dependent increased. In the absence of the plant extract, cercariae showed normal viability without any morphological changes (tail loss) for up to 2 h (Figures 2(a) and 3(a)).

#### 3.1.1. Cercaricidal Activity of *Sida acuta* Hydroethanolic Extract

As shown in Figure 2(b), following 30 min incubation of cercariae with *S. acuta* hydroethanolic extract with 1,000, 500, or 250 *µ*g/mL, 100% of the cercariae died. At the same time, we recorded mortality rates of 83%, 88%, and 91% of cercariae incubated with 125, 62.5, and 31.25 *µ*g/mL of the hydroethanolic extract of *S. acuta,* respectively. However, from 60 min to 150 min, we obtained a mortality rate of 100% with all concentrations of the extract. At 30 minutes time point, the cercaricidal activity of HE*Sa* at all concentrations was significantly higher (*p* < 0.01) than the niclosamide-olamide one (Figure 2(b)).

#### 3.1.2. Cercaricidal Activity of *Sida rhombifolia* Hydroethanolic Extract

The incubation of cercariae with *S. rhombifolia* hydroethanolic extract at 1,000, 500, and 250 *µ*g/mL induced after 30 min, mortality rates of 99%, 97%, and 66%, respectively. At the 60 min time point, we observed a mortality rate of 100% from the cercariae incubated with niclosamide-olamide or HE*Sr* at 1,000 and 500 *µ*g/mL, while the same rate (100%) was obtained after 90 min and 120 min of incubation respectively with 250 and 125 *µ*g/mL of HE*Sr*. The concentrations of 62.5 and 31.25 *µ*g/mL showed a 100% cercariae mortality rate at the 150 min time point. In addition, at 30 minutes time-point, HESr at 500 and 1,000 *µ*g/mL showed a significantly high cercaricidal activity (*p* < 0.001) in comparison to niclosamide one (Figure 3(b)).

#### 3.1.3. Median Lethal Concentration of *Sida acuta* and *Sida rhombifolia* Hydroethanolic Extracts

As shown in Table 1, the cecaricidal median lethal concentration of *S. acuta* extract was almost constant from 60 to 150 mins of incubation. Conversely, the LC_50_ profile was gradually decreased after cercariae incubation with *S. rhombifolia* extract. The LC_50_ of HE*Sa* was then 28.41 ± 3.45 *µ*g/mL and 18.63 ± 0.28 *µ*g/mL after 30 min and 60 min of incubation respectively, while that of HE*Sr* were 172.42 ± 26.16 *µ*g/mL and 56.60 ± 4.07 *µ*g/mL at the same time points. After 150 min of incubation, both extracts disclosed the same LC_50_ (18.15 ± 0.00 *µ*g/mL).

### 3.2. Effect of *Sida acuta* and *Sida rhombifolia* Hydroethanolic Extracts on Mouse Hepatic (Hepa 1–6) Cell Growth

**Figure 4:**
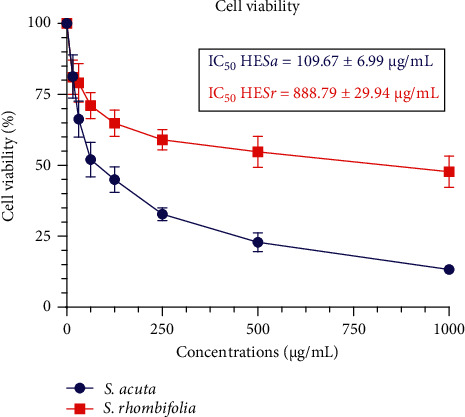
Concentration-response curve of the effect of *Sida acuta* and *Sida rhombifolia* hydroethanolic extracts on Hepa 1–6 cells.

As indicated in Figure 4, the results of the effect of HE*Sa* and HE*Sr* extracts on the growth of the Hepa 1–6 cells at different concentrations (15.625–1,000 *µ*g/mL) showed a decrease in the viability of cells in a concentration-dependent manner after 24 h of incubation.

The inhibitory rates of *S. acuta* and *S. rhombifolia* extracts are shown in Table 2. At 31.25 *µ*g/mL, the inhibitory rate was 28.69% and 14.78% for HE*Sa* and HE*Sr*, respectively. The same tendency was observed at 125 *µ*g/mL where, after 24 h of incubation, inhibitory rates of 51.66% and 29.91% were recorded for HE*Sa* and HE*Sr*, respectively. Furthermore, the incubation of cells with the highest concentration (1,000 *µ*g/mL) shows inhibition rates of 85.69% for HE*Sa* and 51.49% for HE*Sr* (Table 2). Based on their inhibitory activity on the Hepa 1–6 cells, the IC_50_ of HE*Sa* was 109.67 ± 6.99 *µ*g/mL, and that of HE*Sr* was 888.79 ± 29.94 *µ*g/mL.

Based on their cercaricidal activity and their cytotoxicity, the selectivity index of *S. acuta* and *S. rhombifolia* hydroethanolic extracts was calculated. At 30 min post-incubation, this index was 3.86 for HE*Sa* and 5.15 for HE*Sr.*

### 3.3. Phytochemical Constituents of *Sida acuta* and *Sida rhombifolia* Hydroethanolic Extracts

Qualitative phytochemical analysis of hydroethanolic extracts of *S. acuta* (HE*Sa*) and *S. rhombifolia* (HE*Sr*) revealed the presence of some secondary metabolites such as flavonoids, polyphenols, saponins, alkaloids, tannins, anthocyanins, triterpenes, sterols, anthraquinones, coumarins, and cardiac glycosides (Table 3).

**Figure 5:**
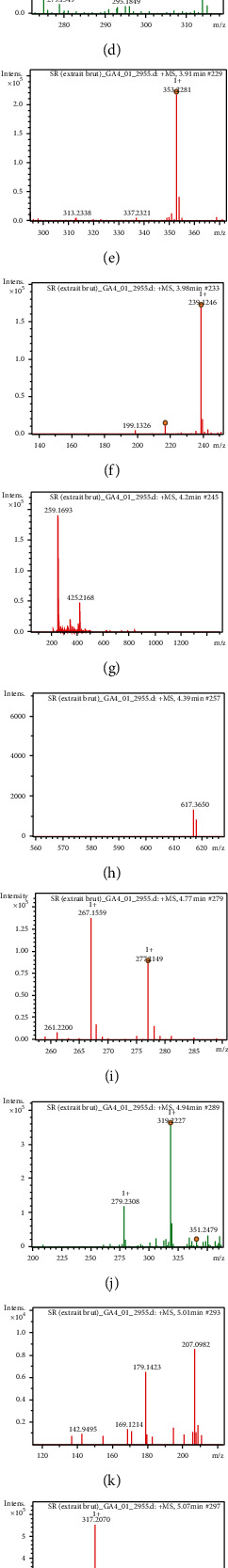
UPLC-DAD-MS UV profile of *Sida rhombifolia* hydroethanolic extract, identified peaks (1–16), and each identified compound spectrum (a–p).

**Figure 6:**
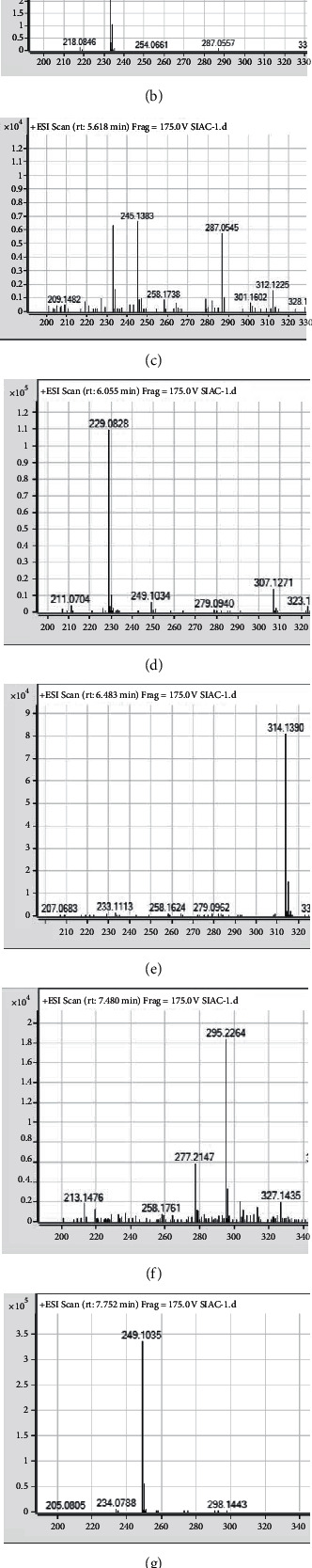
UPLC-DAD-MS UV profile of *Sida acuta* hydroethanolic extract, identified peaks, and each identified compound spectrum (a–i).

The HPLC-MS analysis of *S. rhombifolia* and *S. acuta* hydroethanolic extracts confirmed the presence of alkaloids, polyphenols, and flavonoids. The HPLC-MS chromatogram recorded for HE*Sr* was composed of peaks of chemical compounds, with retention times below 10 min (Figure 5) while those of HE*Sa* were below 15 min (Figure 6). The combination of the MS spectral data and the information from the literature allows tentative identification of 23 compounds (numbered 1–23 on the chromatograms).

The peak eluted at 0.34 min (compound 1), corresponding to the molecular ion [M + Na]+ detected at m/z 203 was credited to glucose with a molecular mass of 203.05 (Figure 5(a)). Compound 2 appears at RT 3.06 min and produced the molecular ion [M + H]+ detected at m/z 219. According to the obtained data, it was possible to infer the molecular mass of 219.09 and identify it as quindoline, an alkaloid (Figure 5(b)). Compound 3 was observed at RT 3.13 min with a molecular ion [M + Na]+ detected at m/z 503 and was identified as 20-hydroxyecdysone, an ecdysteroid with the molecular mass of 503.29 (Figure 5(c)). Concerning compound 4 identification, it appeared at RT 3.50 min with a molecular ion [M + H]+ detected at m/z 314 and was identified as the polyphenol, N-feruloyltyramine with 314.13 as molecular mass (Figure 5(d)). The peak eluted at 4.39 min was allotted to compound 8 with a molecular ion [M + Na]+ at m/z 617 and was identified as tiliroside, a flavonoid with 617.12 as molecular mass (Figure 5(h)). The analysis of the He*Sr* spectrum also allowed to identify two coumarins, thamnosmonin (compound 9, RT 4.77 min with [M + H]+ detected at m/z 277) and scoparone (compound 11, RT 5.01 with [M + H]+ detected at m/z 207) with molecular masses of 277.14 and 207.09, respectively (Figures 5(i) and 5(k)). Moreover, at RT 6.30 min, compound 16 corresponding to the molecular ion [M + Na]+ detected at m/z 413 was identified as di-(2-ethylhexyl) phthalate with the molecular mass of 413.26 (Figure 5(p)). In addition to these identified compounds, compounds 10, 12, 13, 14, and 15 showed at 4.94, 5.07, 5.25, 5.38, and 5.57 min, respectively, were unidentified alkaloids. Also, compounds 5, 6, and 7, with RT 3.91 min, 3.98 min, and 4.20 min, respectively, showed a molecular ion [M + Na]+ at m/z 353, 239, and 259 (Figures 5(e)–5(g)) and were not identified (Table 4).

**Figure 7:**
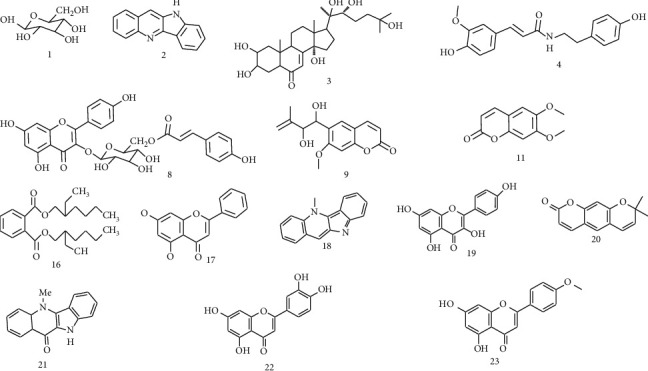
Chemical structures of identified compounds from *Sida acuta* and *Sida rhombifolia* hydroethanolic extracts.

Regarding the HESa chromatogram (Figure 6), the peak eluted at 4.80 min (compound 17), corresponding to the molecular ion [M + Na]+ detected at m/z 277 was credited to chrysin with a molecular mass of 277.09 (Figure 6(a)). Compound 18 appears at RT 5.19 min and produced the molecular ion [M + H]+ detected at m/z 233 and according to the obtained data, it was possible to infer the molecular mass of 219.10 and identify it as cryptolepine, an alkaloid (Figure 6(b)). Compound 19 was observed at RT 5.61 min with a molecular ion [M + H]+ detected at m/z 287 and was identified as kaempferol, a flavonoid with the molecular mass of 287.24 (Figure 5(c)). Concerning compound 20 identification, it appeared at RT 6.05 min with a molecular ion [M + H]+ detected at m/z 229 and was identified as the coumarin, xanthyletin with 229.24 as molecular mass (Figure 6(d)). The peaks eluted at 6.48 min and 7.48 min were N-feruloyltyramine and thamnosmonin corresponding to compounds 4 and 9, respectively. These compounds were previously identified in HE*Sr* (Figures 6(e) and 6(f)). The analysis of the He*Sr* spectrum also allowed to identify two flavonoids, luteolin (compound 22, RT 8.26 min with [M + Na]+ detected at m/z 309) and acacetin (compound 23, RT 12.30 min with [M + Na]+ detected at m/z 284) with molecular masses of 309.24 and 284.26, respectively (Figures 6(h) and 6(i)). Moreover, at RT 7.75 min, compound 21, an alkaloid identified as cryptolepinone was obtained with the molecular ion [M + Na]+ detected at m/z 249 and the molecular mass of 249.28 (Figure 6(g); Table 5). The chemical structures of all identified compounds from *S. acuta* and *S. rhombifolia* hydroethanolic extracts are shown in Figure 7.

## 4. Discussion

The present investigation showed that hydroethanolic (30:70) extracts of *S. acuta* whole plant and *S. rhombifolia* aerial parts disclosed cercaricidal activity against *S. mansoni* cercariae in both time- and concentration-dependent manner. This confirms the larvicidal activity of isolated compounds from *S. rhombifolia* previously highlighted by Islam et al. [23]. Previous studies conducted by some authors have also reported the cercaricidal activity of *Glinus lotoides* fruits aqueous extract [29], *R. vomitoria* stem bark, roots ethanolic extract [7], and *O. pulcherrima* roots methanolic extract and fractions [10]. Other studies have also reported a time- and concentration-dependent molluscicidal, cercaricidal, and/or schistosomicidal activity of various plant extracts such as *A. indica* and *V. amygdalina* [9], *Millettia thonningii* [30, 31], and *Jatropha elliptica* [32]. Based on the median lethal concentration LC_50_ and at any defined period, the cercaricidal activity of *S. acuta* hydroethanolic extract was more important than that of *S. rhombifolia.* As compared with results from previous studies, *S. acuta* and *S. rhombifolia* hydroethanolic 30:70 extracts were more potent, regarding the LC_50_ and the effective time, in killing *S. mansoni* cercariae than *G. lotoides* fruits aqueous extract [29], *R. vomitoria* stem bark and roots ethanolic extracts [7], *A. indica* and *V. amygdalina* methanolic extracts [9] but less effective than *O. pulcherrima* roots methanolic extract [10]. The cercaricidal activity of *S. acuta* and *S. rhombifolia* extracts might be due to their secondary metabolites such as flavonoids, alkaloids, and saponins. Abo-Zeid and Shohayeb [8] have shown the anti-miracidial and anti-cercarial activities of total alkaloids, saponins, and volatile oil extracted from *N. sativa* seeds hydroethanolic extract. It has also been demonstrated that alpinumisoflavone and robustic acid, two isoflavonoids isolated from *M. thonningii* seeds exhibit *in vitro* cercaricidal activity [30, 31]. Moreover, Dos Santos et al. [32] reported a strong cercaricidal activity of diethyl 4-phenyl-2,6-dimethyl-3,5 pyridine dicarboxylate, a penta-substituted pyridine alkaloid from the rhizome of *J. elliptica* with an LC_100_ of 2 *µ*g/mL. The cercaricidal potential of these secondary metabolites could strongly support the efficacy of *S. acuta* and *S. rhombifolia* extracts in killing *S. mansoni* cercariae. Indeed, phytochemicals belonging to flavonoids, polyphenols, coumarins, alkaloids, and steroids were isolated from *S. rhombifolia* hydroethanolic 30:70 extract in this study. The cercaricidal activity of *S. acuta* and *S. rhombifolia* may be due to their ability to damage the cercariae tegument and disturb its motor activity. Xiao et al. [33] have previously demonstrated that exposure of *S. japonicum* cercariae to praziquantel results in intensive disturbance in motor activity and lysis of cercarial tissues, followed by an extensive release of gland contents and separation of the tail from the body. These observations were followed by the cercariae surface damages that are characterized by the decrease of the membranous glycocalyx, the swelling, and the degeneration of mitochondria distributed in the muscle and parenchymal cells as well as the lysis of the tegumental muscular layer. In fact, in our study, the incubation of *S. mansoni* cercaria with *S. acuta* and *S. rhombifolia* hydroethanolic extracts resulted in the separation of their tail from their body.

The genus *Sida* L. is one of the most diverse in the Malvaceae family, with about 200 species scattered in every part of tropical and subtropical regions in the world [18, 34, 35]. In addition, 142 chemical constituents belonging to various classes have been reported for *Sida* sp. Alkaloids, flavonoids, and ecdysteroids were predominant and reported mostly from *S. acuta*, *S. cordifolia*, *S. rhombifolia*, *S. glutinosa*, and *S. spinosa*. [36]. In our study, alkaloids were the major bioactive principles of *S. acuta* and *S. rhombifolia* hydroethanolic 30:70 extracts. Moreover, among the 15 compounds identified in those extracts, only thamnosmonin and xanthyletin (coumarins), and tiliroside (flavonoid) were recently identified in *S. rhombifolia* hydroethanolic extract [37]. Some of these compounds have been previously identified and/or isolated from the *Sida* L. genus. In fact, quindoline has been isolated from *S. cordifolia* and *S. rhombifolia* [38, 39], while 20-hydroxyecdysone has been isolated from *S. spinosa* [40] *S. cordifolia* [41], *S. tuberculata* [42–44], and *S. acuta* [37]. Furthermore, N-feruloyltyramine and di-(2-ethylhexyl) phthalate have been isolated from *S. acuta* [45] and scoparone from *S. rhombifolia* [46]. Moreover, Das et al. [47] identified chrysin in *S. glutinosa* and Silva et al. [48] isolated luteolin from *S. galheirensis.* Acacetin and kaempferol were isolated from *S rhombifolia* [38, 39], while cryptolepine and cryptolepinone were characterized from *S. acuta* [38, 39]. These phytoconstituents exhibited various pharmacological activities such as anti-microbial, anti-plasmodial, vasorelaxant, anti-oxidant, and anti-inflammatory [38, 42, 44, 49, 50].

Regarding the cytotoxic activity of both extracts on the growth of the Hepa 1–6 cells, *S. acuta* extract disclosed the highest anti-proliferative activity than *S. rhombifolia*. Anti-proliferative activity of a plant extract may be linked to its phytochemical constituents. It has been shown that quindoline and cryptolepinone, as well as N-trans-feruloyltyramine identified in *S. acuta* and *S. rhombifolia* hydro ethanolic extracts have a significant anti-proliferative activity on mouse hepatoma cells (Hepa 1c1c7) by inhibiting the quinone reductase activity [46].

## 5. Conclusions

*S. acuta* and *S. rhombifolia* hydroethanolic extracts disclosed cercaricidal activity and were not toxic on Hepa 1–6 cell line. However, Based on the LC_50_, the cercaricidal activity was more pronounced with *S. acuta* extract. The cercaricidal activity of these plant extracts may be linked to the presence of several secondary metabolite such as alkaloids, flavonoids, and coumarins. Based on their activity and their selectivity index, *S. rhombifolia* extract could be more effective on S. mansoni cercariae than S. acuta extract. This study could provide baseline information for further investigations aiming to develop plant-based alternative drugs against *S. mansoni*.

## Figures and Tables

**Table 1:** LC_50_ values of *Sida acuta* and *Sida rhombifolia* hydroethanolic extracts on *Schistosoma mansoni* cercariae at different time points.

LC_50_ (*µ*g/mL)		
Time (min)	HE*Sa*	HE*Sr*
30	28.41 ± 3.45	172.42 ± 26.16
60	18.63 ± 0.28	56.60 ± 4.07
90	18.74 ± 0.59	32.29 ± 4.05
120	18.15 ± 0.00	21.95 ± 1.54
150	18.15 ± 0.00	18.15 ± 0.00

**Table 2:** Inhibition rates of the *Sida acuta* and *Sida rhombifolia* hydroethanolic extracts on the growth of the Hepa 1–6 cells.

Percentage of inhibition		
Concentrations	HE*Sa*	HE*Sr*
15.625 *µ*g/mL	12.42 ± 1.54	12.42 ± 0.54
31.25 *µ*g/mL	28.69 ± 1.33	14.78 ± 0.73
62.5 *µ*g/mL	44.20 ± 2.33	23.11 ± 1.33
125 *µ*g/mL	51.66 ± 1.12	29.91 ± 0.91
250 *µ*g/mL	64.53 ± 1.21	36.12 ± 1.41
500 *µ*g/mL	75.62 ± 1.80	41.11 ± 1.44
1000 *µ*g/mL	85.69 ± 0.41	51.49 ± 0.45

**Table 3:** Secondary metabolites of *Sida acuta* and *Sida rhombifolia* hydroethanolic extracts.

Phytochemicals	HE*Sa*	HE*Sr*
Anthocyanins	+	−
Triterpenes	+	+
Sterols	+	−
Flavonoids	+	+
Polyphenols	+	+
Saponins	+	+
Essential oils	−	−
Anthraquinones	+	−
Alkaloids	+	+
Tannins	−	+
Coumarins	+	+
Reducing sugar	−	+
Cardiac glycosides	+	+

(+): presence; (−): absence.

**Table 4:** Main signals exhibited in the LC-DAD-MS spectra of compounds detected in the hydroethanolic extract from *Sida rhombifolia* aerial parts and proposed attribution.

RT (min)	Exp. mass	Cald. mass	Molecular formula	Identified compounds	Structure
0.34	203.0527; [M + Na]^+^	203.0526	C_6_H_12_O_6_Na	Glucose (sugar)	**1**
3.06	219.0986; [M + H]^+^	219.0922	C_15_H_11_N_2_	Quindoline (alkaloid)	**2**
3.13	503.2959; [M + Na]^+^	503.2979	C_27_H_44_O_7_Na	20-Hydroxyecdysone (steroid)	**3**
3.50	314.1371; [M + H]^+^	314.1387	C_18_H_20_NO_4_	N-Feruloyltyramine (polyphenol)	**4**
3.91	353.2281; [M + Na]^+^	353.2298	C_20_H_34_O_5_Na	NI	5
3.98	239.1246; [M + Na]^+^	239.1254	C_11_H_20_O_4_Na	NI	6
4.20	259.1693; [M + Na]^+^	259.1669	C_15_H_24_O_2_Na	NI	7
4.39	617.1259; [M + Na]^+^	617.1271	C_30_H_26_O_13_Na	Tiliroside (flavonoid)	**8**
4.77	277.1409; [M + H]^+^	277.0895	C_15_H_17_O_5_	Thamnosmonin (coumarin)	**9**
4.94	319.2227; [M + H]^+^	319.2227	C_15_H_31_N_2_O_5_	NI (alkaloids)	10
5.01	207.0982; [M + H]^+^	207.0657	C_11_H_11_O_4_	Scoparone (coumarin)	**11**
5.07	463.2637; [M + Na]^+^	463.2626	C_19_H_40_N_2_O_9_	NI (alkaloid)	12
5.25	363.2484; [M + H]^+^	363.2490	C_17_H_35_N_2_O_6_	NI (alkaloid)	13
5.38	635.4454; [M + Na]^+^	635.4453	C_30_H_64_N_2_O_10_Na	NI (alkaloid)	14
5.57	377.2641; [M + H]^+^	377.2646	C_18_H_37_N_2_O_6_	NI (alkaloid)	15
6.30	413.2643; [M + Na]^+^	413.2663	C_24_H_38_O_4_Na	Di-(2-ethylhexyl) phthalate	**16**

NI: not identified.

**Table 5:** Main signals exhibited in the LC-DAD-MS spectra of compounds detected in the hydroethanolic extract from *Sida acuta* the whole plant and proposed attribution.

RT (min)	Exp. mass	Cald. mass	Molecular formula	Identified compounds	Structure
4.80	277.0977; [M + Na]^+^	277.24	C_15_H_10_O_4_Na	Chrysin (flavonoid)	**17**
5.19	233.1090; [M + H]^+^	233.28	C_16_H_12_N_2_	Cryptolepine (alkaloid)	**18**
5.61	287.0545; [M + H]^+^	287.24	C_15_H_10_O_6_	Kaempferol (flavonoid)	**19**
6.05	229.0828; [M + H]^+^	229.24	C_14_H_13_O_3_	Xanthyletin (coumarin)	**20**
6.48	314.1371; [M + H]^+^	314.13	C_18_H_21_NO_4_	N-Feruloyltyramine (polyphenol)	**4**
7.48	277.2147; [M + H]^+^	277.14	C_11_H_20_O_4_	Thamnosmonin (coumarin)	**9**
7.75	249.1035; [M + H]^+^	249.28	C_16_H_13_N_2_O	Cryptolepinone (alkaloid)	**21**
8.26	309.2067; [M + Na]^+^	309.24	C_15_H_10_O_6_Na	Luteolin (flavonoid)	**22**
12.30	284.2629; [M + Na]^+^	284.26	C_16_H_12_O_5_Na	Acacetin (flavonoid)	**23**

## Data Availability

The data used to support the findings of this study are available from the corresponding author upon request.
